# Bivalent and Monovalent SARS-CoV-2 Variant Vaccine Boosters Improve coverage of the known Antigenic Landscape: Results of the COVID-19 Variant Immunologic Landscape (COVAIL) Trial

**DOI:** 10.21203/rs.3.rs-2653179/v1

**Published:** 2023-05-05

**Authors:** Angela Branche, Nadine Rouphael, David Diemert, Ann Falsey, Cecilia Losada, Lindsey (R) Baden, Sharon Frey, Jennifer Whitaker, Susan Little, Evan Anderson, Emmanuel Walter, Richard Novak, Richard Rupp, Lisa Jackson, Tara Babu, Angelica Kottkamp, Annie Luetkemeyer, Lilly Immergluck, Rachel Presti, Martin Backer, Patricia Winokur, Siham Mahgoub, Paul Goepfert, Dahlene Fusco, Elissa Malkin, Jeff Bethony, Edward Walsh, Daniel Graciaa, Hady Samaha, Amy Sherman, Stephen Walsh, Getahun Abate, Zacharoula Oikonomopoulou, Hana El Sahly, Thomas Martin, Satoshi Kamidani, Michael Smith, Benjamin Ladner, Laura Porterfield, Maya Dunstan, Anna Wald, Tamia Davis, Robert Atmar, Mark Mulligan, Kirsten Lyke, Christine Posavad, Megan Meagher, David Stephens, Kathleen Neuzil, Kuleni Abebe, Heather Hill, Jim Albert, Kalyani Telu, Jinjian Mu, Teri Lewis, Lisa Giebeig, Amanda Eaton, Antonia Netzl, Sam Wilks, Sina Tureli, Mamodikoe Makhene, Sonja Crandon, David Montefiori, Mat Makowski, Derek Smith, Seema Nayak, Paul Roberts, John Beigel

**Affiliations:** University of Rochester; Emory Unviversity; George Washington University; University of Rochester; Emory University Hope Clinic; Brigham & Women’s Hospital and Harvard Medical School; Saint Louis University; Baylor College of Medicine; Department of Medicine, University of California, San Diego, CA 92903; Duke University School of Medicine; University of Illinois at Chicago; University of Texas Medical Branch; Kaiser Permanente Washington Health Research Institute; University of Washington; NYU Langone Manhattan; Zuckerberg San Francisco General Hospital UCSF; Morrehouse School of Medicine; Washington University School of Medicine; NYU Langone Long Island; University of Iowa; Howard University Hospital; Department of Medicine, Division of Infectious Diseases, University of Alabama at Birmingham; George Washington University; George Washington University; University of Rochester; Department of Medicine, Emory University School of Medicine; Emory University School of Medicine; Brigham and Women's Hospital; Harvard University; Saint Louis University; Saint Louis University; Baylor College of Medicine; University of California San Diego; Center for Childhood Infections and Vaccines (CCIV) of Children's Healthcare of Atlanta and Emory University Department of Pediatrics; Duke University; University of Illinois at Chicago; University of Texas Medical Branch; Kaiser Permanente Washington Health; University of Washington; NYU Langone Manhattan; Baylor College of Medicine; NYU Langone Medical Center; Center for Vaccine Development and Global Health, University of Maryland School of Medicine; Department of Laboratory Medicine and Pathology, University of Washington; Fred Hutchinson Cancer Center; Emory University; University of Maryland School of Medicine; NIH; The Emmes Company; The Emmes Company, LLC; The Emmes Company; The Emmes Company; 29. Clinical Monitoring Research Program Directorate, Frederick National Laboratory for Cancer Research; 29. Clinical Monitoring Research Program Directorate, Frederick National Laboratory for Cancer Research; Duke University Medical Center; University of Cambridge; Centre for Pathogen Evolution, Department of Zoology, University of Cambridge; University of Cambridge; Division of Microbiology and Infectious Diseases, National Institute of Allergy and Infectious Diseases (NIAID), National Institutes of Health (NIH); Division of Microbiology and Infectious Diseases, NIAID, NIH; Duke; The Emmes Company; University of Cambridge; Division of Microbiology and Infectious Diseases, NIAID, NIH; Division of Microbiology and Infectious Diseases, National Institute of Allergy and Infectious Diseases (NIAID), National Institutes of Health (NIH); Leidos Biomedical Research Inc.

## Abstract

Vaccine protection against COVID-19 wanes over time and has been impacted by the emergence of new variants with increasing escape of neutralization. The COVID-19 Variant Immunologic Landscape (COVAIL) randomized clinical trial (clinicaltrials.gov NCT 05289037) compares the breadth, magnitude and durability of antibody responses induced by a second COVID-19 vaccine boost with mRNA (Moderna mRNA-1273 and Pfizer-BioNTech BNT 162b2), or adjuvanted recombinant protein (Sanofi CoV2 preS DTM-AS03) monovalent or bivalent vaccine candidates targeting ancestral and variant SARS-CoV-2 spike antigens (Beta, Delta and Omicron BA.1). We found that boosting with a variant strain is not associated with loss in neutralization against the ancestral strain. However, while variant vaccines compared to the prototype/wildtype vaccines demonstrated higher neutralizing activity against Omicron BA.1 and BA.4/5 subvariants for up to 3 months after vaccination, neutralizing activity was lower for more recent Omicron subvariants. Our study, incorporating both antigenic distances and serologic landscapes, can provide a framework for objectively guiding decisions for future vaccine updates.

## BACKGROUND

Severe Acute Respiratory Syndrome Coronavirus 2 (SARS-CoV-2) has infected over 750 million people worldwide and resulted in more than 6 million deaths, including more than 1 million deaths in the United States (US).^1,2^ The COVID-19 vaccines authorized for emergency use or fully approved in the US are safe and highly effective against severe disease and death.^3-6^ However, vaccine protection against symptomatic infection wanes over time. ^7-10^ Additionally, new variants of concern (VOCs) have repeatedly emerged, including B.1.351 (Beta), B.1.617.2 (Delta), B.1.1.529 (Omicron BA.1) and Omicron subvariants, all with mutations in the spike protein receptor binding domain (RBD) that result in diminished viral neutralization by antibodies,^11-13^ leading to increased rates of infections. While additional booster doses of the ancestral strain improve vaccine effectiveness against VOCs in the short term,^14-20^ variant-specific boosters may optimize vaccine immunogenicity against current and future VOCs.

In this adaptive phase 2 clinical trial, we evaluated boosting with ancestral and variant SARS-CoV-2 spike protein(s) (Beta, Delta and Omicron BA.1), alone or in combination, using both mRNA vaccines (Moderna and Pfizer BioNTech mRNA), and recombinant protein vaccine (Sanofi AS03-adjuvanted), to assess the breadth, magnitude and durability of neutralizing antibody responses.

## RESULTS

### Study Population

From March 30 to May 6, 2022, 602 participants were randomized and 597 received a Moderna mRNA vaccine in stage 1 (Table 1, Supplemental Figure S15).^21^ From May 12 to 27, 2022, 313 participants were randomized and 312 received a Pfizer BioNTech mRNA vaccine in stage 2 (Table 1 and Supplemental Figure S16). From June 8 to 17, 2022, 153 participants were randomized and 152 received a Sanofi protein vaccine in stage 3 (Table 1 and Supplemental Figure S17). Baseline demographics were similar across between study arms within each stage (Table 1). Median age was 53 years (range: 18–85) for stage 1, 47 years (range: 20–83) for stage 2 and 45 years (range: 18–79) for stage 3; 35%, 30% and 20% were ≥ 65 years for each stage, respectively. The majority of participants were female (53–59%); 6 to 11% were Hispanics and 73 to 82% were White. Most participants (94–100% per arm) had received an mRNA-based primary series and initial boost vaccination. Twenty percent, 33% and 41% in stages 1, 2 and 3, respectively, were defined as previously infected based on anti-N antibody seropositivity at baseline and/or by self-reported past positive SARS-CoV-2 PCR or antigen testing. Median duration (range) between study vaccination and the last previous vaccination or infection was 168 (110–333) days, 198 (106–333) days and 197 (79–359) days for stages 1, 2 and 3, respectively. Median follow up duration at data cutoff was 228 days, 193 days and 176 days for stages 1, 2 and 3 respectively.

### Safety

The frequency and severity of solicited local and systemic adverse events (AEs) after vaccination were similar to other booster trials^22^ and did not differ between arms in each stage (Supplemental Figs. 1–6). The most frequently reported solicited local AE was injection-site pain (83% of participants for stage 1, 77% for stage 2, 74% for stage 3). The most common solicited systemic AEs were fatigue (50–67%) and myalgia (39–57%). Most solicited AEs were mild to moderate with only 0–1% severe local AEs and 0.7-4% severe systemic AEs. A summary of all AEs is presented in Supplemental Figs. 7–9. As of the data cutoff, 13 participants in stage 1, 4 participants in stage 2, and 1 participant in stage 3 had a serious AE (SAE); all were deemed unrelated to study product. There was one related AESI in stage 1 of a young man who reported chest pain 1 day after vaccination that was initially evaluated as possible myocarditis, which was ultimately excluded due to a normal troponin I level and normal cardiac MRI. There was one death unrelated to study product due to cardiac arrest from advanced coronary artery disease.

### Neutralizing Antibody Responses for Stage 1 (Moderna mRNA)

Stage 1 participants were boosted with either the Moderna mRNA-1273 ancestral (Prototype) vaccine or one of five different variant-targeting vaccine products including monovalent BA.1, and bivalent vaccines comprised of BA.1 and either B.1.351 (Beta), B.1.617.2 (Delta) or ancestral (Prototype) spike (Table 1). BA.1 was the omicron variant vaccine available at the start of this trial. Neutralizing antibodies (PsVN Ab) were assessed against pseudoviruses expressing the spike proteins of ancestral (D614G) SARS-CoV-2 and variants B.1.617.2, B.1.351, BA.1 and BA.4/5 at baseline and on Days 15, 29 and 91.

For stage 1, PsVN Ab responses peaked at Day 15 after vaccination, remained relatively stable at Day 29, were similar between older (≥ 65 years) and younger adults, and were 2–3 times higher in previously infected compared to uninfected participants (Supplemental Tables 7 and 8). PsVN Ab geometric mean titers (GMTs) against all variants declined from Day 29 to Day 91 by a factor of 1.74 (95% CI: 1.69, 1.80) in previously uninfected participants and by a factor of 1.34 (95% CI: 1.25, 1.44) in previously infected participants (p < 0.001) (Supplemental Fig. 10).

**Figure 1:**
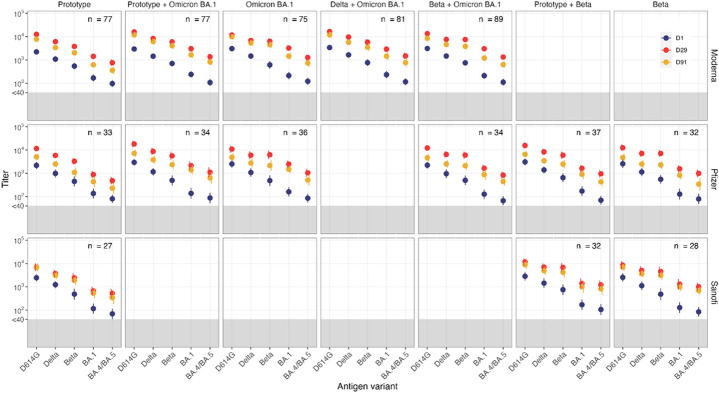
Pseudovirus Neutralization ID_50_ Titers by Timepoint (Baseline, Day 29 and Day 91) and Variant (D614G, Delta, Beta, Omicron BA.1 [B.1.1.529] and Omicron BA.4/BA.5) in Uninfected Participants by vaccine arm and platform. Circles denote GMT, geometric mean titer with 95% CI. GMT at pre-vaccination baseline, obtained on Day 1 are presented in blue and post-vaccination Day 29 GMT and Day 91 GMT in red and yellow, respectively.

For uninfected participants, all Omicron BA.1 containing vaccines (Day 29 GMT_D614G_ between 11,963 and 16,001) boosted PsVN Ab to D614G similarly to the Prototype vaccine (Day 29 GMT_D614G_= 12,600). (Fig. 1) The Prototype vaccine was less effective in boosting against all variants, based on point estimates (GMT_B.1.617.2_=6,181; GMT_B.1.351_=3,535; GMT_BA.1_=1,343; GMT_BA.4/5_= 722 at Day 29) when compared to variant vaccines (GMT_B.1.617.2_ between 6,902 and 9,342; GMT_B.1.351_ between 5,744 and 7,016; GMT_BA.1_ between 2,684 and 3,005; GMT_BA.4/5_ between 1,190 and 1,384 at Day 29). In particular, monovalent or bivalent Omicron BA.1 vaccines did not differ numerically in their ability to neutralize all variants tested (Supplemental Fig. 11, Supplemental Table 7). The geometric mean fold rises (GMFR) at Day 29 in all Omicron BA.1 containing vaccines against Omicron variants (GMFR_BA.1_= 11.6 to 14.6 and GMFR_BA.4/5_=10.6 to 12.7) and B.1.351 (GMFR_B.1.351_=7.7 to 10.1) were higher when compared to B.1.617.2 (GMFR_B.1.617.2_=5.0 to 6.7) or D614G (GMFR_D614G_=4.3 to 5.7) suggesting either differences in antibody maturation or antigenic distance among the variants versus a ceiling with D614G.

The antibody responses with Omicron BA.1-containing vaccines were more durable, with a smaller geometric mean fold decline (GMFD) from Day 29 to Day 91 for Omicron subvariants (GMFD_BA.1_=2.0 to 2.2 and GMFD_BA.4/5_= 1.8 to 2.0) and B.1.351 (GMFD_B.1.351_= 1.4 to 1.7) when compared to Prototype vaccine (GMFD_BA.1_= 2.3, GMFD_BA.4/5_=2.1, GMFD_B.1.351_=1.8). Within each study arm, the ratio in geometric mean neutralization titer against variant pseudoviruses compared to the ancestral D614G pseudovirus (GMR_D614G_) was used as a measure of boosting effect, where lower values correspond to stronger responses of variant vaccines to variants other than D614G. GMR_D614G_ values also reflect the extent of neutralization escape, where higher values correspond to greater escape. In stage 1, less immune escape from Omicron variants was observed for Omicron BA.1-containing vaccines (GMR_D614G_= 7.13 to 8.72 for BA.1 and 13.40 to 16.13 for BA.4/5) than with the Prototype vaccine (GMR_D614G_= 12.0 for BA.1 and 20.6 for BA.4/5) at Day 91 (Supplemental Fig. 12, Supplemental Table 7).

### Neutralizing Antibody Responses for Stage 1 Against Additional Omicron Subvariants

**Figure 2:**
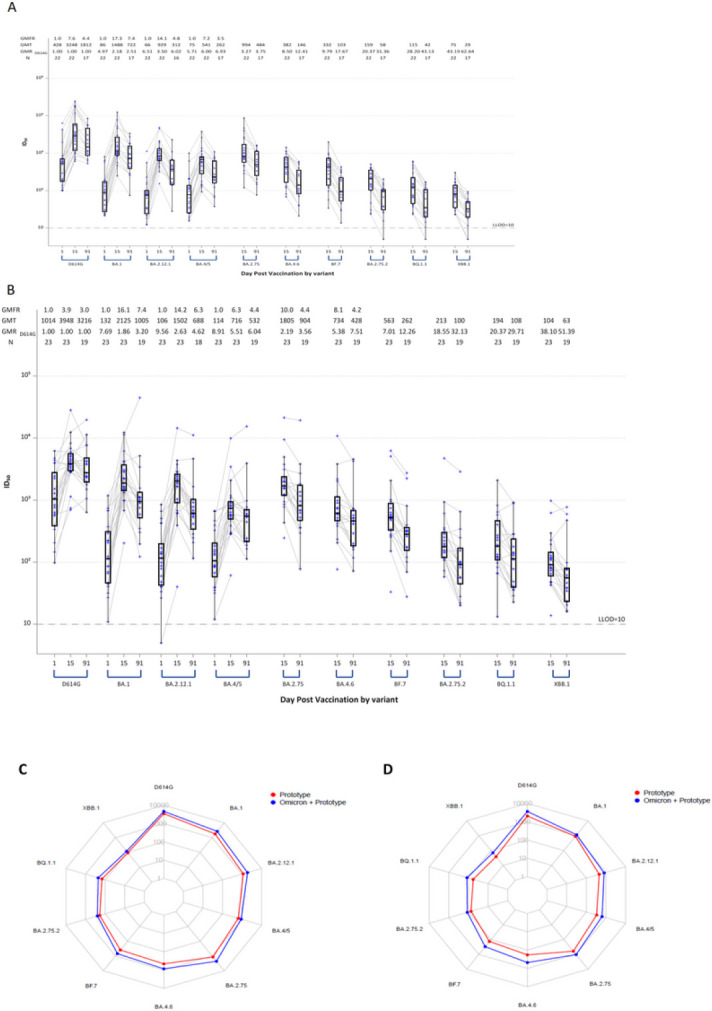
Pseudovirus Neutralization ID_50_ Titers by Timepoint (Day 1, Day 15 and Day 91) and Variant (D614G, Omicron BA.1, BA.2.12.1, BA.4/BA.5, BA.2.75, BA.4.6, BF.7, BA.2.75.2, BQ.1.1 and XBB.1) in a Subset (N = 22-23) of Uninfected Participants: A) Stage 1 Moderna mRNA-1273 Prototype monovalent vaccine. Boxes and horizontal bars denote interquartile range (IQR) and median ID_50_, respectively. Whisker denotes 95% confidence interval. LLOD, lower limit of detection of the assay. GMT, geometric mean titer. GMR_D614G_, geometric mean ratio against D614G. N, number of samples tested. B) Stage 1 Moderna mRNA-1273 Omicron BA.1 + Prototype bivalent vaccine. Boxes and horizontal bars denote interquartile range (IQR) and median ID_50_, respectively. Whisker denotes 95% confidence interval. LLOD, lower limit of detection of the assay. GMT, geometric mean titer. GMR_D614G_, geometric mean ratio against D614G. N, number of samples tested. C) Radar plots of the pseudovirus neutralization GMTs at Day 15 for the two vaccine arms in Stage 1 Moderna mRNA-1273 Prototype monovalent vaccine (red) and Moderna mRNA-1273 Omicron BA.1 + Prototype bivalent vaccine (blue). Circles are GMT estimates for each variant. D) Radar plots of the pseudovirus neutralization GMTs at Day 91 for the two vaccine arms in Stage 1 Moderna mRNA-1273 Prototype monovalent vaccine (red) and Moderna mRNA-1273 Omicron BA.1 + Prototype bivalent vaccine (blue). In the radar plots, each variant is represented by its own vertical line or spoke, and the spokes are evenly distributed around the circle. Each horizontal line along a vertical spoke represents the Geometric Mean Titer (GMT) at a 10-fold dilution with the value closest to the center being 1 and farthest from the center being 10,000 or 10^4^. A line is drawn connecting the GMT data values for vaccine arm at the individual variants represented by its vertical spoke.

Serum samples from a subset of 22–23 uninfected participants in stage 1 who were boosted with either the Moderna monovalent Prototype vaccine or the Moderna bivalent Omicron BA.1 + Prototype vaccine were tested at Day 15 and Day 91 for PsVN Ab to D614G and Omicron subvariants BA.1, BA.2.75, BA.2.12.1, BA.4/5, BA.4.6, BF.7, BA.2.75.2, BQ.1.1 and XBB.1 (Fig. 2 and Supplemental Table 13). The assays were performed in a separate laboratory using a pseudovirus platform that resembles but is not identical to the one used for the other datasets in this study.

PsVN Ab were highest against the ancestral D614G variant in both groups. Higher GMT estimates against all Omicron subvariants were observed at Day 15 with the Omicron BA.1 + Prototype bivalent vaccine when compared to the Prototype. More pronounced immune escape was seen with the recent variants (BQ.1.1 and XBB.1). The PsVN Ab response was more durable with the bivalent compared to the Prototype vaccine at Day 91 relative to Day 15 with a GMFD of 2.8 and 2.7 for the Prototype vaccine compared to a decline of 2.1 and 1.9 for the Omicron BA.1 + Prototype vaccine against BQ.1.1 and XBB.1, respectively.

These results highlight the remarkable speed at which the Omicron lineage evolved to evade vaccine-elicited neutralizing antibodies, where recent subvariants (e.g., BQ.1.1, XBB.1) are substantially more resistant than earlier subvariants (e.g., BA.1, BA.2.75) regardless of whether the BA.1 spike was present in the vaccine boost. The results also suggest modest improvement in durability of serum neutralizing antibodies against all variants after bivalent vaccine boosting compared to prototype vaccine boosting.

### Neutralizing Antibody Responses for Stage 2 (Pfizer-BioNTech mRNA)

Stage 2 participants were boosted with either the Pfizer BNT162b2 Wildtype vaccine or one of five different variant-targeting versions of Pfizer BNT162b2 COVID-19 vaccine, including monovalent BA.1, monovalent B.1.351, a bivalent BA.1 + Wildtype vaccine, and two additional bivalent vaccines comprised of B.1.351 and either BA.1 or Wildtype spike (Table 1). Neutralizing antibodies were assessed with the same assay used for the main dataset in stage 1.

Consistent with stage 1 results involving a similar mRNA vaccine technology, PsVN Ab peaked on Day 15, remained relatively stable on Day 29, were similar between older (≥ 65 years) and younger adults, and were 2–4 times higher in previously infected participants compared to uninfected participants (Supplemental Tables 9 and 10, Figs. 1 and Supplemental Figs. 10).

For uninfected participants (Supplemental Table 9, Supplemental Fig. 11), all variant-containing vaccines (either Beta or Omicron BA.1) boosted D614G PsVN Ab (Day 29 GMT_D614G_ between 10,951 and 18,093) similarly to the Wildtype vaccine (Day 29 GMT_D614G_= 11,600). As in stage 1, the Wildtype vaccine was less effective in boosting against all variants (GMT_B.1.617.2_=5,890; GMT_B.1.351_=3,313; GMT_BA.1_ =888; GMT_BA.4/5_= 485 at Day 29) when point estimates were compared to all other variant vaccines (GMT_B.1.617.2_ between 6,002 and 8,721; GMT_B.1.351_ between 5,664 and 6,253; GMT_BA.1_ between 1,411 and 2,480; GMT_BA.4/5_ between 839 and 1,054 at Day 29).

B.1.351 contains far fewer spike mutations than BA.1 and BA.4/5; however, all three variants share a common set of mutations in the receptor binding domain (K417N, E484K/A, N501Y). These three mutations alone might account for the modestly improved neutralizing antibody responses against Omicron seen with the Beta and Beta + Wildtype vaccines compared to the Wildtype monovalent vaccine. However, while monovalent Omicron BA.1 and monovalent Beta vaccines similarly boosted titers to the B.1.351 variant (GMT_B.1.351_ of 6,253 and 6,247 respectively), they numerically differed in their ability to neutralize Omicron BA.1 (GMT_BA.1_ of 2,480 and 1,411 respectively).

The GMFRs at Day 29 for all variant vaccines against Omicron (GMFR_BA.1_= 9.5 to 17.3 and GMFR_BA.4/5_=11.4 to 14.2) and B.1.351 variants (GMFR_B.1.351_=9.1 to 13.9) were higher when compared to B.1.617.2 (GMFR_B.1.617.2_=5.5 to 8.3) or D614G variants (GMFR_D614G_=4.3 to 7.0). Of note, for the Wildtype vaccine, GMFRs for the variants tested were not numerically different and ranged between 5.3 and 7.3.

More durable antibody responses were observed with most variant-targeting vaccines, with a smaller GMFD from Day 29 to Day 91, particularly for Omicron subvariants (GMFD_BA.1_= 1.6 to 2.0 and GMFD_BA.4/5_= 1.6 to 2.4) when compared to Wildtype vaccine (GMFD_BA.1_ = 2.1, GMFD_BA.4/5_ = 2.1). Additionally, compared to responses against D614G, less immune escape to Omicron variants was observed for variant-containing vaccines (GMR_D614G_= 3.3 to 7.1 for BA.1 and 9.5 to 15.1 for BA.4/5) than with the Wildtype vaccine (GMR_D614G_= 11.6 for BA.1 and 22.1 for BA.4/5) at Day 91. (Supplemental Fig. 12 and Supplemental Table 9)

### Neutralizing Antibody Responses for Stage 3 (Sanofi AS03-adjuvanted protein)

Stage 3 participants were boosted with one of three Sanofi adjuvanted recombinant spike protein vaccine products, including the prototype vaccine, a monovalent Beta vaccine, and a bivalent Beta + Prototype vaccine (Table 1). No product containing Omicron spike protein was available at the time the study was conducted and Day 15 samples were not tested for stage 3 arms. Neutralizing antibodies were assessed on Days 1, 29 and 91 in the same assay used for the main datasets in stages 1 and 2.

PsVN Ab at Day 29 after vaccination with Sanofi variant vaccines were similar between older (≥ 65 years) and younger adults. Day 29 PsVN Ab titers were approximately 4–5 times higher in previously infected compared to uninfected recipients of the monovalent Beta and monovalent Protytpe vaccines arms but there was less numerical difference in Day 29 titers (1.5-2 times higher) between previously infected and uninfected recipients of the bivalent Beta + Prototype vaccine. (Fig. 1, Supplemental Tables 11 and 12 and Supplemental Fig. 10).

For uninfected participants, all Beta-containing vaccines boosted D614G antibody titers (Day 29 GMT_D614G_ between 9,384 and 11,726) as well as the Prototype vaccine (Day 29 GMT_D614G_= 6,942) (Supplemental Fig. 11, Supplemental Table 11). The Prototype vaccine was less effective in boosting against most variants (GMT_B.1.617.2_=3,739; GMT_B.1.351_=2,437; GMT_BA.1_ =667 at Day 29) when compared to the two variant vaccines (GMT_B.1.617.2_ between 5,670 and 6,996; GMT_B.1.351_ between 5,173 and 6,785; GMT_BA.1_ between 1,169 and 1,391 at Day 29) based on point estimates. The GMFRs from baseline to Day 29 in both variant vaccines against Omicron (GMFR_BA.1_= 7.7 to 12.0 and GMFR_BA.4/5_=9.2 to 10.3) and B.1.351 (GMFR_B.1.351_=8.6 to 16.3) and B1.617.2 (GMFR_B.1.617.2_=4.7 to 8.9) variants were numerically higher when compared to D614G variants (GMFR_D614G_=4.0 to 7.0).

Similar or more durable antibody responses were seen in PsVN Ab titers from Day 29 to Day 91 with Beta-containing vaccines against Omicron subvariants (GMFD_BA.1_= 1.5 to 2.1 and GMFD_BA.4/5_= 1.5 to 1.7) when compared to Prototype vaccine (GMFD_BA.1_= 1.5, GMFD_BA.4/5_= 2.0). Additionally, compared to responses against D614G, less immune escape by Omicron variants was observed for the Beta + Prototype vaccine (GMR_D614G_= 9.1 for BA.1 and 11.6 for BA.4/5) than with the Prototype vaccine (GMR_D614G_= 13.1 for BA.1 and 21.5 for BA.4/5) at Day 91, based on point estimates, although confidence intervals overlapped (Supplemental Table 11 and Supplemental Fig. 12).

### ANCOVA Geometric Mean Ratio of Variant Containing Vaccines to Prototype/Wildtype Vaccines

In ANCOVA models for each stage, the Day 91 geometric mean ratio (GMR) comparing neutralization titers with variant-containing vaccines to first generation Prototype/Wildtype vaccines against the ancestral D614G variant ranged from 0.85–1.58 f for each variant vaccine within the 3 stages.

In stage 1, all Omicron BA.1-containing Moderna vaccines led to a Day 91 GMR_BA.1_≥ 1.88, GMR_BA.4.5_≥ 1.7 and GMR_B1.351_ ≥1.5 compared to the Prototype vaccine, with unadjusted lower bound confidence intervals > 1 (Supplemental Table 14). In stage 2, all Omicron BA.1- or Beta-containing Pfizer vaccines led to a Day 91 GMR_BA.1_≥ 1.99, GMR_BA.4.5_≥ 1.8 and GMR_B.1.351_ ≥1.78 compared to the Wildtype vaccine (Supplemental Table 15). The Day 91 GMRs in stages 1 and 2 were similar or higher to those observed for D29. In stage 3, all Beta-containing Sanofi vaccines led to a Day 91 GMR of greater than 1 relative to the Prototype vaccine, though the unadjusted lower bound confidence interval did cross 1 (Supplemental Table 16).

### Antigenic Cartography and Antibody Landscapes

**Figure 3:**
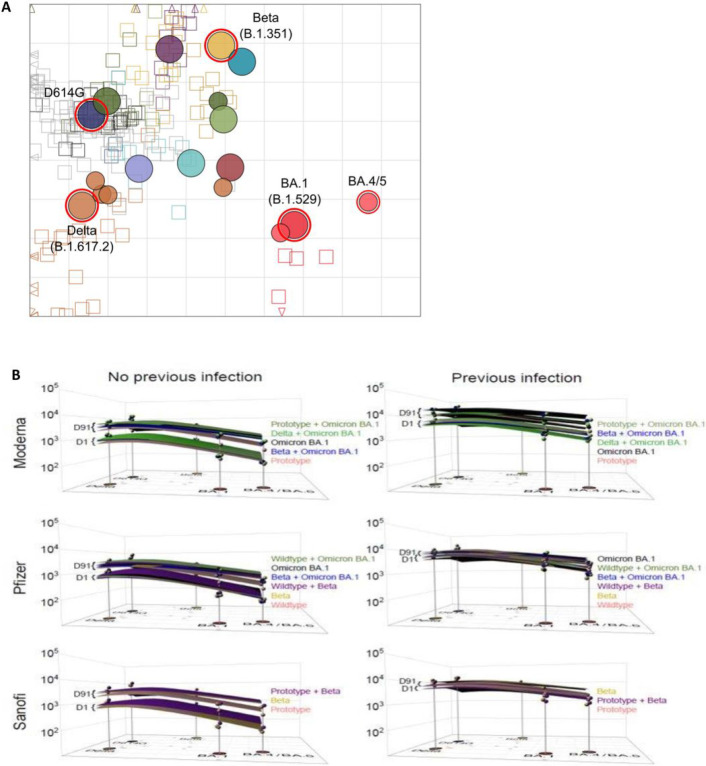
Antigenic Cartography. A) An adapted version of the antigenic map by Wilks, et al.^22^ served as base map for all antibody landscapes. Virus variants are shown as filled circles, variants with additional substitutions from their root variant as smaller circles. Individual sera are displayed as open squares in the color of their root variant or grey for mRNA-1273 vaccinated sera, small dark squares represent clinical trial participants. One grid unit in the map corresponds to a two-fold dilution in the neutralization assay, within x- and y-axis the map orientation is free as antigenic distances are relative. Small triangles point to sera outside the shown map area. B) Day 1 and Day 91 geometric mean titer (GMT) antibody landscapes for uninfected and infected individuals in different arms for the 3 stages. Impulses show the GMT against the specific variant. Lower landscapes correspond to Day 1 and upper landscapes to Day 91 immunity. To interpret landscapes, a Day 91 response where upper landscape is flat, indicates the responses to all the variants were equivalent, whereas, skewing up or down indicates an uneven response across variants. The surface colors represent study arms (Red: Prototype, Light Green: Prototype + Omicron BA.1, Black: Omicron BA.1, Dark Green: Delta + Omicron BA.1, Blue: Beta + Omicron BA.1, Purple: Beta + Prototype, Yellow: Beta).

The antigenic landscapes for each vaccine arm across the 3 stages (Fig. 3A) were derived based on the antigenic map base map by Wilks et. al.^23^. Figure 3B shows the GMT antibody landscapes for each vaccine arm in the 3 stages stratified by prior infection, with the corresponding neutralizing antibody titers above the variant’s map position.

All vaccine arms for each of their respective stages in uninfected participants had similar pre-vaccination antigenic landscapes, with the apex over D614G, as expected (Fig. 3B). After vaccination, all arms, in all 3 stages, had antibody titers that raised and flattened the antigenic landscape. In uninfected cohorts for all 3 stages, variant-containing vaccines lifted titers against BA.1 and BA.4/5 and flattened the landscapes better than the Prototype or Wildtype vaccines. A second booster dose raised antibody titers in uninfected participants to the titers observed in previously infected participants at baseline (Fig. 3B) (Supplemental Figs. 13 and 14).

### SARS-CoV-2 infections

There were 267 self-reported COVID-19 illnesses, occurring after randomization among 973 participants in single dose arms by data cutoff, 1 of which resulted in a brief hospitalization, lasting less than 24 hours, due to hypoxemia. The incidence of infections in this trial reflect the community transmission, with the majority occurring during the Omicron BA.5 wave in the United States. At any point in time, participants from different stages will be in different points in follow up. Therefore assessing incidence across stages is not a valid comparison. Kaplan-Meier (KM) estimates of infections at the latest timepoint were similar among arms within a stage. (Supplemental Tables 4–6) A higher percentage of infections, across all stages, was noted in participants with no history of prior infection (KM estimate: 37.8% [95% CI: 31.8%,44.6%]) compared to those with a history of prior infection, documented by self-report and/or N-antibody positivity (KM estimate: 12.1% [95%CI: 8.4%,17.2%]). There were also fewer infections in adults ≥ 65 years (KM estimate: 19.3% [95% CI: 15.1%,24.5%]) compared to their younger counterparts (KM estimate: 36.2% [95%CI: 29.2%,44.4%]) across all stages.

## DISCUSSION

The continued emergence of SARS-CoV-2 VOCs led to a recommendation to update COVID-19 vaccines.^24^ The strains selected in 2022 for modified vaccines covered circulating strains at the time of vaccine development, not necessarily variants that would drift antigenically from Omicron BA.1 and BA.4/5 or evolve from other distinct locations on the phylogenetic tree. Therefore, it is important to investigate not only immune responses to known variants, but also the antigenic relationships among different SARS-CoV-2 VOCs^25^ and how variant vaccines may alter immunologic landscapes to cover antigenic areas where new strains may emerge. Here, we described the magnitude, breadth, and landscapes of the neutralizing antibody response following a second booster with investigational monovalent and bivalent variant-specific vaccines reflective of the diverse SARS-CoV-2 immunologic background seen in the general population and utilizing different vaccine platforms.

Our findings support that mRNA and adjuvanted protein variant vaccines elicit substantial cross-reactive neutralizing antibodies to D614G as well as to B.1.351, 1.617.2, Omicron BA.1, Omicron BA.4/5 and other Omicron subvariants, regardless of prior SARS-CoV-2 infection history and age. This is likely due to ongoing antibody somatic mutation, memory B cell clonal turnover and development of antibodies that are resistant to SARS-CoV-2 Spike protein receptor binding domain (RBD) mutations.^26,27^

Secondly, the mRNA variant vaccines offered a clear serologic advantage over the Wildtype/Prototype vaccines against B.1.351, BA.1 and BA.4/5 that persisted up to 3 months after vaccination. Moreover, vaccine candidates without Omicron BA.1 variant, such as the Pfizer mRNA Beta vaccine, still provided superior heterologous coverage to Omicron BA.1 and BA.4/5 when compared to the Wildtype vaccine, which was likely due to the common mutations in the spike RBD (K417N, E484K/A, N501Y) between B.1.351 and these Omicron variants. While a serologic advantage to BA.1 was not seen in ANCOVA modelling with the Sanofi Beta or Beta + Prototype protein vaccine candidates, perhaps due to small sample size or undetected prior infection, a similar serologic benefit of boosting with the Beta monovalent vaccine,^28,29^ as well as superior clinical efficacy against Omicron BA.1 and BA.2, was seen in the manufacturer’s phase 3 clinical trial.^30^

The antibody landscapes visualizing the neutralization profile after vaccination further support inclusion of variants in booster vaccines. After vaccination, the antibody landscape rises with variant vaccine candidates, especially against more recent variants, and flattens the antibody landscape more than the Prototype vaccine, suggesting there may be higher titers of neutralizing antibodies with variant-containing vaccines against future VOCs especially if they emerge near B.1.351, Omicron BA.1 and BA.4/5.^31^

Though specific correlates of protection for infection with recent Omicron subvariants are not well understood, neutralizing antibody titers have been used to infer protection during the D614G wave of the pandemic, when the circulating virus closely matched the vaccine strain,^32^ and the resulting immunologic data has served as the basis for emergency use authorization for booster vaccines by regulatory agencies.^33,34^ The improved serologic response with variant containing vaccines over Prototype/Wildtype vaccines in our study and others^35-37^provide evidence that broad cross-protection may be conferred without a variant-chasing approach and warrants further mechanistic exploration.

For all vaccine candidates, including vaccine products not containing Prototype, the antibody titers were higher against D614G compared to the VOCs, supporting the hypothesis of back-boosting to the ancestral strain seen in previous studies.^36-38^ This suggests that future generations of SARS-CoV-2 vaccines may be able to omit Prototype or Wildtype sequences without losing the ability to neutralize D614G, or other variants within close antigenic distance, in people who previously received the Prototype vaccines. Furthermore, Omicron BA.1 or Beta monovalent vaccines were nearly equivalent to Omicron BA.1 + Prototype or Beta + Prototype bivalent vaccines for neutralization of B.1.351 and both Omicron subvariants (BA.1 and BA.4/5) further supporting the premise that monovalent variant vaccines could replace bivalent vaccines as the updated boost in the future.^30^

Notably, although variant vaccines improved neutralizing activity against Omicron subvariants, these titers decreased for more recent Omicron subvariants. While the ID_50_ against BA.1 and BA.4/5 remained high, the neutralization titers for subvariants BQ.1.1 and XBB.1 were much lower. Additionally, we noted a high rate of infections which occurred during the BA.4/5 wave and subsequent waves with XBB.1 and BQ.1.1. These infections occurred more frequently in previously uninfected compared to previously infected individuals highlighting the importance of hybrid immunity in protection against disease.^31^ In addition, infections occurred in younger rather than older adults likely reflecting behavioral differences impacting risk of exposure. Our study was not designed to assess vaccine effectiveness (VE). Though recent data suggest possibly higher VE against Omicron subvariants with bivalent vaccine boosts (Prototype + Omicron BA.4/5, Prototype + Omicron BA.1) compared with the Prototype vaccine,^18,39^ our findings highlight concerns that variant vaccines are unlikely to keep pace with virus evolution and that other immune correlates of protection beyond antibody responses need to be explored.

Our study has several limitations. First, the sample size is small for certain subgroups of interest such prior infection (27%) and adults older than 65 years (31%). Second, T cell responses and antibody effector functions, which may be critical to preventing severe disease,^40^ have not yet been evaluated. Additionally, clonal and kinetic analyses of the memory B cell response, while underway, are not available to further differentiate the durability of the antibody response elicited by variant-containing vaccines. Finally, participants were only randomized to different arms within each stage and not between stages which enrolled sequentially at different calendar times leading to different exposures to circulating variants prior to and after enrollment. This precludes head-to-head comparisons of rates of infections or neutralization titers across stages.

In conclusion, these data demonstrate that updating vaccines to target recent variants provides modestly improved and broadly cross-protective neutralizing antibody responses against diverse SARS-CoV-2 variants without sacrificing boosting immunity to the ancestral strain. The precise degree to which the enhanced antibody response elicited by updated vaccines will restore protection against disease after infection with heterologous or homologous strains needs further confirmation by real-world effectiveness studies. Our study incorporating both antigenic distances and serologic landscapes serve as a framework for objectively guiding decisions for future vaccine updates.

## METHODS

### Study Design and Eligibility Criteria

This phase 2 open-label, randomized, clinical trial was performed at 22 sites in the US (Supplemental Table 1). Eligible participants were healthy adults 18 years of age and older (with or without a history of prior SARS-CoV-2 infection) who had received a primary series and a single homologous or heterologous boost with an approved or emergency use authorized COVID-19 vaccine (Supplemental Table 2). The most recent vaccine dose, and/or prior infection must have occurred at least 16 weeks prior to randomization. Full eligibility criteria are described at clinicaltrials.gov (NCT 05289037).

Eligible participants were stratified by age (18–64 and ≥ 65 years) and history of confirmed SARS-CoV-2 infection, and randomly assigned across arms within each stage in an equal ratio using block randomization methodology with blocks of size 6 and 12 for stages 1 and 2 and blocks of size 3 and 6 for stage 3. Subjects were randomized using the Advantage eClinical system used by the Statistical Data Coordinating Center. As this was an unblinded study, no effort was made to conceal the assignment post randomization. Sample size was chosen to be able to detect common adverse events and estimate immunogenicity parameters with acceptable precision (See protocol for further details). After providing informed consent, participants underwent screening, including confirmation of COVID-19 vaccination history, medical history, a targeted physical examination, and a urine pregnancy test (if indicated). Safety and immunogenicity assessments were performed on Days 1, 15 and 29, and at 3, 6, 9 and 12 months after last vaccination. Although the study was not designed to evaluate booster vaccine effectiveness, we collected information on antigen or PCR-confirmed symptomatic or asymptomatic SARS-CoV-2 infection at any time after randomization. A nasal swab sample was collected for viral sequencing in persons testing positive. Immunologic data are currently available up to the Day 91 visit after first vaccination. The safety data cutoff was December 2, 2022.

The trial was reviewed and approved by a central institutional review board and overseen by an independent Data and Safety Monitoring Board. The trial was sponsored and funded by the National Institutes of Health (NIH). The NIAID SARS-CoV-2 Assessment of Viral Evolution (SAVE) program team was consulted to inform study arm design and variant vaccine selection.

### Trial vaccines

Trial vaccines are listed in Table 1 and Supplemental Table 3. Trial vaccines were provided by Moderna (Cambridge, MA) for stage 1 (50 mcg per vaccine), Pfizer BioNTech (New York, NY) for stage 2 (30 mcg per vaccine) and Sanofi (Paris, France) for stage 3 (5 mcg per vaccine). The vaccine candidates were manufactured similarly to their corresponding authorized or approved vaccines in the US or Europe.

### Study outcomes

The primary objective was to evaluate humoral immune responses of candidate SARS-CoV-2 variant vaccines, alone or in combination. The secondary objective was to evaluate the safety of candidate SARS-CoV-2 variant vaccines assessed by solicited injection site and systemic adverse events (AEs), which were collected for 7 days after vaccination; unsolicited AEs through Day 29; and serious adverse events (SAEs), new-onset chronic medical conditions (NOCMCs), adverse events of special interest (AESIs), AEs leading to withdrawal, and medically attended adverse events (MAAEs) through the duration of the trial.

Exploratory objectives included sequencing strains from infections for variant spike lineage and assessing anti-nucleocapsid serology. Information on antigen- or PCR- confirmed symptomatic or asymptomatic SARS-CoV-2 infection at any time after randomization was collected.

### Immunogenicity assays

SARS-CoV-2 neutralization titers, expressed as the serum inhibitory dilution required for 50% neutralization (ID_50_), were assessed using pseudotyped lentiviruses presenting SARS-CoV-2 spike mutations for the D614G, (Wuhan-1 containing a single D614G spike mutation), B1.617.2 (Delta), B.1.351 (Beta) and B.1.1.529 (Omicron BA.1) variants, as described previously.^22,41^ A random subset of samples (25 per selected vaccine arms, distributed equally between age strata and sites) were analyzed for neutralization titers to the Omicron BA.4/BA.5, BA.2.12.1, BA.2.75.2, BA.2.75, BA.4.6, BF.7 and BQ.1.1 subvariants in a separate laboratory.^42^ Electrochemiluminescence immunoassays (ELECSYS) were used for the detection of anti-nucleocapsid (N) (N-ELECSYS; Elecsys Anti-SARS-CoV-2 N, Roche, Indianapolis) at baseline.^41^

### Statistical analysis

The primary objective of this study is to evaluate the magnitude, breadth and durability of SARS-CoV-2 specific antibody titers in serum samples by estimating 95% confidence intervals (CI) for the geometric mean titer (GMT) at each timepoint when samples are collected. No pre-specified formal hypothesis tests were planned. The geometric mean fold rise (GMFR) is calculated as the geometric mean of titers at a timepoint divided by titers at Day 1. The geometric mean ratio to D614G (GMR_D614G_) is the geometric mean of the ratio of D614G titers against titers for a variant of concern. Seropositive rate is calculated as the proportion of participants with titers above the lower limit of detection (LLOD). 95% CI for GMT, GMFR, and GMR_D614G_ are calculated using the Student’s t-distribution and 95% CI for seropositive rate is calculated using the Clopper-Pearson binomial method. For the purpose of analysis, participants were defined as previously infected by self-report of a confirmed positive antigen or PCR testing or the detection of anti-SARS-CoV-2 N antibodies. Participants with a SARS-CoV-2 infection occurring between vaccination and a pre-specified immunogenicity timepoint were excluded from immunogenicity analysis at that timepoint and thereafter.

ANCOVA models were used to estimate GMT ratios of variant vaccines compared to Prototype vaccine and included independent variables for vaccination arms, age (18–64 years and ≥ 65 years of age), previous infection history, and baseline titers. For modeling purposes, titers were log_10_ transformed and estimated mean differences were back transformed to generate GMT ratios between vaccination groups. Unadjusted 97.5% confidence intervals based on the t-distribution are reported.

Infection rates are estimated using Kaplan-Meier methodology.

All analyses were done in SAS v9.4 or R v4.2.2 or higher.

### Antigenic cartography and antibody landscapes

Antigenic cartography uses antibody neutralization data to position virus variants and sera relative to each other in an n-dimensional Euclidean space, in this case a 2-dimensional space, as previously described.^43,44^ The distance between variants can be understood as a measure of antigenic similarity. Briefly, for each serum-variant pair, the fold-change from the maximum titer variant in the specific serum is calculated to obtain a target distance from the serum. Serum and variant coordinates are then optimized such that difference between Euclidean map distance and this target distance is minimized, with one map unit corresponding to one two-fold dilution of neutralization titers on the log2 scale. Here, the antigenic map published in Wilks *et al.*^44^ was used as basis for the antibody landscapes, where neutralization titers against virus variants are plotted in a third dimension above the corresponding variant in an antigenic map and a continuous surface is fitted to these titers.^23^ Antibody landscapes were constructed using the ablandscape.fit function^45,46^ of the ablandscape package (v = 1.1.0, R v = 4.2.0) with the parameters method = “cone”, error.sd = 1, bandwidth = 1, degree = 1, control = list(optimise.cone.slope = TRUE). Variant coordinates from the base map were used to fit a single-cone surface to neutralization titers against D614G, B.1.351, B.1.617.2, BA.1 and BA.4/5 for each serum. Per arm, the surface slope was optimized to match pre- and 3 months post-vaccine neutralization titers. Samples from non-responding participants, defined as a titer of 20 (LLOD/2) against all variants at either time point were not included (n = 12 in the uninfected cohort, n = 3 in the infected cohort).

## Data Availability

All data is included in the manuscript. The protocol for the study is provided as supplementary materials.

## Code availability

The code for antibody landscapes and titer line plots is publicly available in a GitHub repository (https://github.com/acorg/branche_et_al2023).
